# Supplementary material for: The Effects of Insulin on Immortalized Rat Schwann Cells, IFRS1
Source: Int J Mol Sci. 2021 May 23;22(11):5505. doi: 10.3390/ijms22115505 (PMC8197103; doi:10.3390/ijms22115505)
Supplement: Supplementary file 1 [file ijms-22-05505-s001.zip › ijms-1163062-supplementary.pdf]

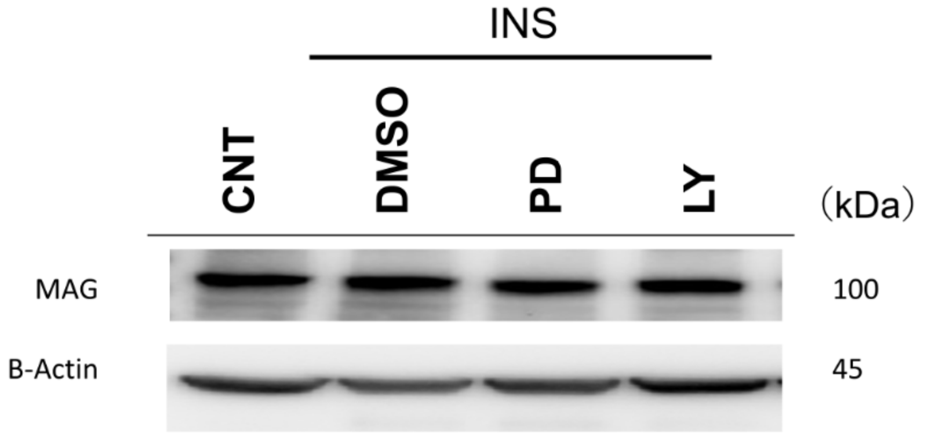

Figure S1. Western blot data showing the expression of MAG through insulin stimulation, and the effect of LY294002 and PD98059 on MAG.
